# Complex hybrid repair of multifocal visceral and aortoiliac pseudoaneurysms associated with neurosarcoidosis

**DOI:** 10.1016/j.jvscit.2026.102501

**Published:** 2026-09-04

**Authors:** Mauricio Gonzalez-Urquijo, Jack Thorburn, Mahdi Baba, Jason Bayne, Daniel Obrand, Elie Girsowicz

**Affiliations:** Division of Vascular Surgery, McGill University, Montreal, QC, Canada

**Keywords:** Neurosarcoidosis, Visceral pseudoaneurysm, Visceral artery, Hybrid approach

## Abstract

We report the case of a 36-year-old man with neurosarcoidosis who developed multiple pseudoaneurysms of the superior mesenteric artery (SMA), infrarenal aorta, and bilateral iliac arteries. Fluorodeoxyglucose-positron emission tomography demonstrated no significant uptake within the aneurysmal segments. Given the complex vascular anatomy, a staged hybrid approach was undertaken. Initial treatment consisted of a hepatic artery-to-distal SMA bypass followed by plug embolization of the SMA aneurysms. Subsequently, aortoiliac reconstruction was performed with a left aortouni-iliac endograft, femoral-femoral bypass, and right external-to-internal iliac artery stenting. This case highlights a rare vascular manifestation of sarcoidosis.

Sarcoidosis is a multisystem granulomatous disease of unclear etiology characterized by noncaseating granulomatous inflammation that most commonly affects the lungs, lymphatic system, skin, and nervous system.^1^ Neurosarcoidosis represents a relatively uncommon manifestation, occurring in approximately 5% to 15% of patients with systemic sarcoidosis.^2^ Vascular involvement in sarcoidosis is rare but has been increasingly recognized, ranging from large-vessel aortitis to peripheral vasculitis and aneurysmal degeneration.^3^ Reports of multifocal visceral and aortoiliac pseudoaneurysms associated with neurosarcoidosis remain exceptionally uncommon. We present a complex case of a young patient with neurosarcoidosis who developed multiple visceral and aortoiliac pseudoaneurysms requiring staged hybrid vascular reconstruction.

Informed consent has been obtained from the patient for publication of the case report and accompanying images.

## Case presentation

A 36-year-old man with a history of previous cigarette smoking (approximately one pack daily for 5 years), current cannabis use of approximately three joints daily, previous cocaine use, and occasional alcohol consumption was admitted with several months of progressive bilateral lower-extremity weakness and numbness. The patient denied intravenous drug use. Family history was negative for aneurysmal disease, collagen vascular disorders, rheumatologic disease, neurologic disease, or autoimmune conditions. Extensive neurologic investigations demonstrated widespread spinal cord inflammation. Initial investigations revealed a positive interferon gamma release assay, and spinal tuberculosis was initially suspected (Fig 1). However, cerebrospinal fluid analysis obtained following lumbar puncture was negative, and the patient failed to respond to empiric antituberculous therapy. Following extensive multidisciplinary evaluation, including neuroimaging, cerebrospinal fluid analysis, exclusion of alternative infectious and inflammatory etiologies, and multiple nondiagnostic tissue biopsies, the patient fulfilled the 2018 Neurosarcoidosis Consortium Consensus Group criteria for possible neurosarcoidosis. The patient was subsequently treated with corticosteroids and maintenance infliximab therapy every 4 weeks. During hospitalization, the patient developed cauda equina syndrome as a complication following lumbar puncture, resulting in persistent lower-extremity weakness and neurogenic bladder and bowel dysfunction with urinary and fecal incontinence. The patient was also diagnosed with bilateral renal cell carcinoma and underwent right nephrectomy and left partial nephrectomy 3 months after his admission.

**Fig 1:**
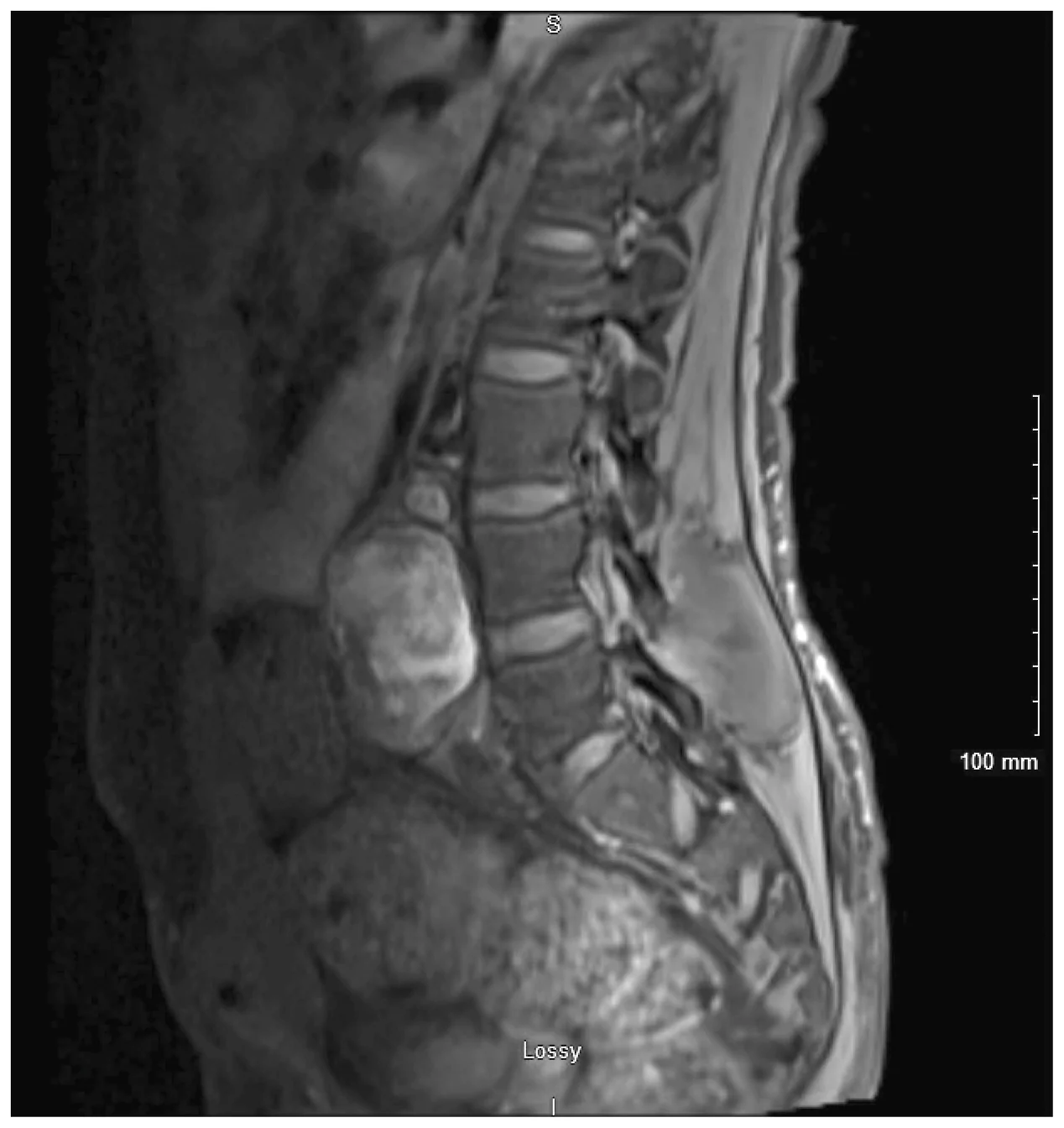
Sagittal contrast-enhanced T1-weighted magnetic resonance imaging (MRI) of the lumbar spine demonstrating inflammatory changes centered at the L4 to L5 level, with vertebral endplate destruction, abnormal enhancement of the adjacent vertebral bodies and intervertebral disc, and an associated prevertebral/paravertebral soft-tissue collection suspicious for tuberculosis.

The patient was discharged to a rehabilitation facility on postoperative day 30. He subsequently represented on multiple occasions with recurrent fevers of unclear origin. Initial treatment included empiric oral antibiotics for presumed urinary tract infection, later escalated to broad-spectrum antimicrobial therapy. Anti-tumor necrosis factor-α therapy was temporarily withheld. Repeated infectious investigations failed to identify a definitive source of infection. Fluorodeoxyglucose (FDG)-positron emission tomography (PET) imaging performed 2 months after his discharge demonstrated multiple aneurysmal lesions involving several vascular territories without significant FDG uptake, including tandem superior mesenteric artery (SMA) pseudoaneurysms measuring 3.3 × 3.9 and 2.6 × 3.0 cm, a saccular infrarenal abdominal aortic pseudoaneurysm measuring 2.3 × 3.9 cm, a right common iliac artery pseudoaneurysm measuring 5.8 × 4.2 cm, a left iliac artery pseudoaneurysm measuring 2.1 × 2.2 cm (Fig 2).

**Fig 2:**
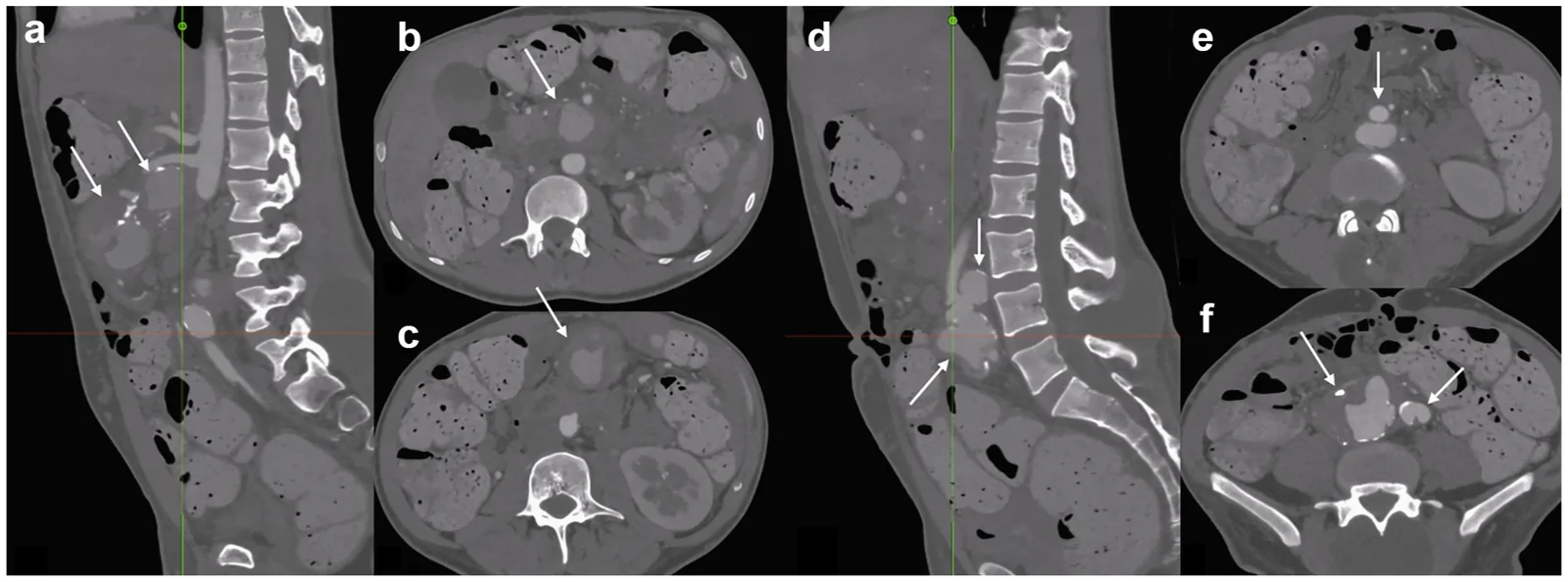
**a,** Sagittal view demonstrating two tandem superior mesenteric artery pseudoaneurysms. **b,** Axial view showing the proximal superior mesenteric artery pseudoaneurysm measuring 3.3 × 3.9 cm. **c,** Axial view showing the distal superior mesenteric artery pseudoaneurysm measuring 2.6 × 3.0 cm. **d,** Sagittal view demonstrating an infrarenal abdominal aortic pseudoaneurysm located below the origin of the inferior mesenteric artery, and right common iliac pseudoaneurysm. **e,** Axial view of the abdominal aortic pseudoaneurysm measuring 2.3 × 3.9 cm. **f,** Axial view showing a right common iliac artery pseudoaneurysm measuring 5.8 × 4.2 cm and a left common iliac artery pseudoaneurysm measuring 2.1 × 2.2 cm. *White arrows* pointing towards pseudoaneurysms.

Before the intervention, the patient underwent multidisciplinary evaluation by vascular surgery, neurology, rheumatology, and infectious diseases. At the time of surgery, he was receiving corticosteroids and maintenance infliximab therapy, and FDG-PET demonstrated no evidence of active vascular inflammation within the aneurysmal segments. He subsequently underwent staged hybrid vascular intervention. Initial treatment consisted of visceral revascularization with a hepatic artery-to-distal SMA branch bypass using a 6-mm Propaten graft (W. L. Gore & Associates) via a transperitoneal approach. Although distal SMA perfusion remained intact, the distal main SMA was unsuitable for direct reconstruction because of the extent of aneurysmal involvement and the absence of an adequate disease-free segment for anastomosis. Therefore, a healthy distal SMA branch was selected as the outflow target. This was followed by endovascular exclusion of the SMA pseudoaneurysms using 16- and 12-mm Amplatzer Vascular Plug II devices (Abbott) deployed through a transfemoral percutaneous approach (Fig 3). Owing to operative complexity, the abdomen was temporarily left open using an ABThera Open Abdomen Negative Pressure Therapy System (3M), with a planned second-look laparotomy the following day. The patient returned to the operating room the following day for re-exploration, which demonstrated preserved bowel viability and satisfactory hemostasis, permitting abdominal closure. Postoperatively, the patient developed hemoperitoneum requiring exploratory laparotomy, which revealed no identifiable source of active bleeding.

**Fig 3:**
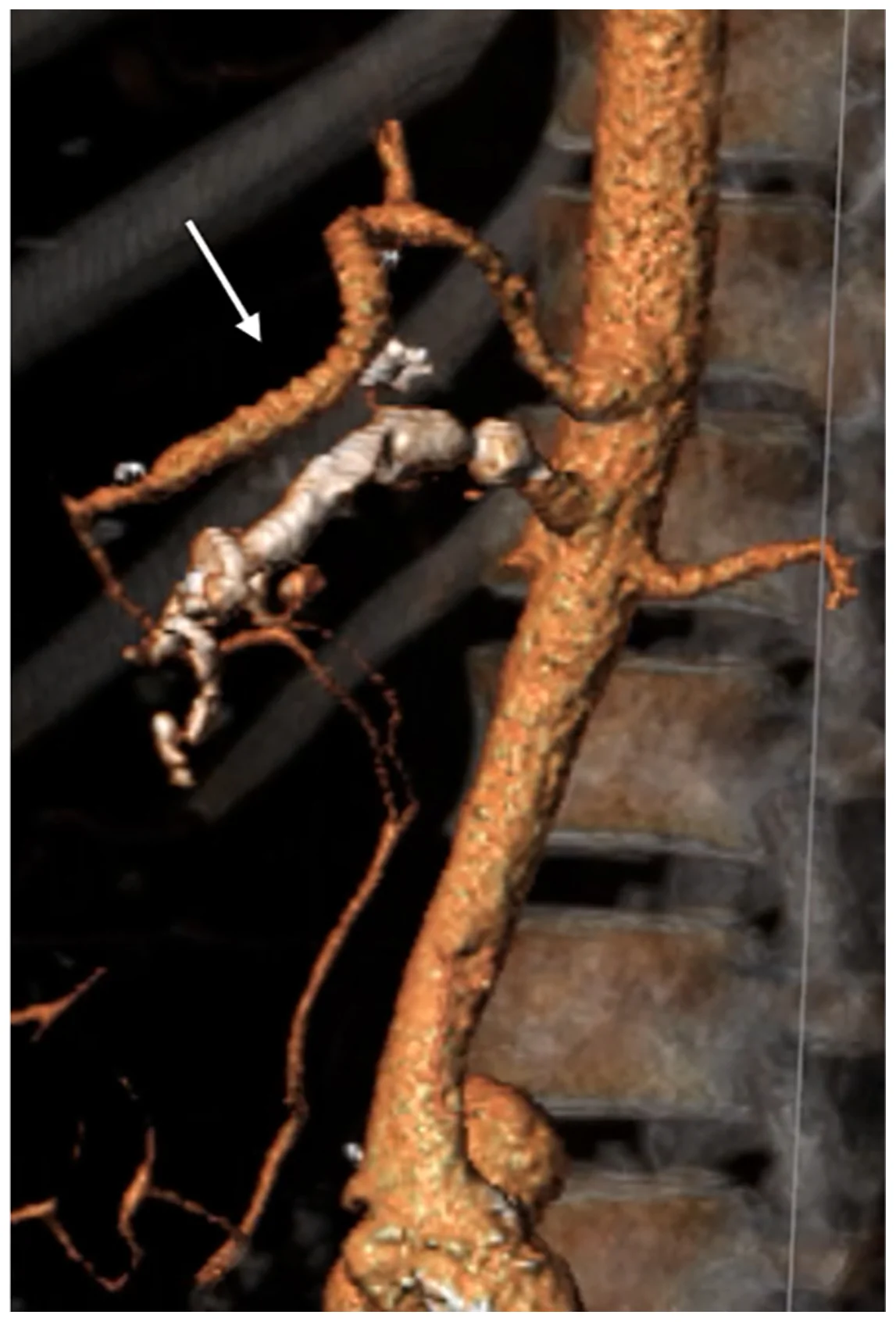
Three-dimensional (3D) reconstruction of computed tomography angiography showing hepatic artery-to-distal superior mesenteric artery branch bypass using a 6-mm Propaten graft (W. L. Gore & Associates). *White arrow* pointing towards the graft.

Fourteen days later, the patient underwent staged treatment of the infrarenal aortoiliac aneurysmal disease using a hybrid approach. Endovascular repair was performed with a left aortouni-iliac reconstruction using a 16 × 13 × 93 mm Endurant II iliac limb endograft (Medtronic) deployed immediately below the inferior mesenteric artery to preserve mesenteric perfusion (Fig 4). The right internal iliac artery was preserved using an 11 mm × 10 cm VIABAHN covered stent (W. L. Gore & Associates) extending from the external to the internal iliac artery according to a “banana” configuration technique, excluding the right common iliac aneurysm (Fig 5). Revascularization of the right lower extremity was achieved with a left-to-right femoral-femoral crossover bypass using an 8-mm prosthetic graft tunneled subcutaneously. Completion angiography confirmed exclusion of the aneurysmal segments with preserved aortoiliac and pelvic perfusion. No major perioperative complications occurred.

**Fig 4:**
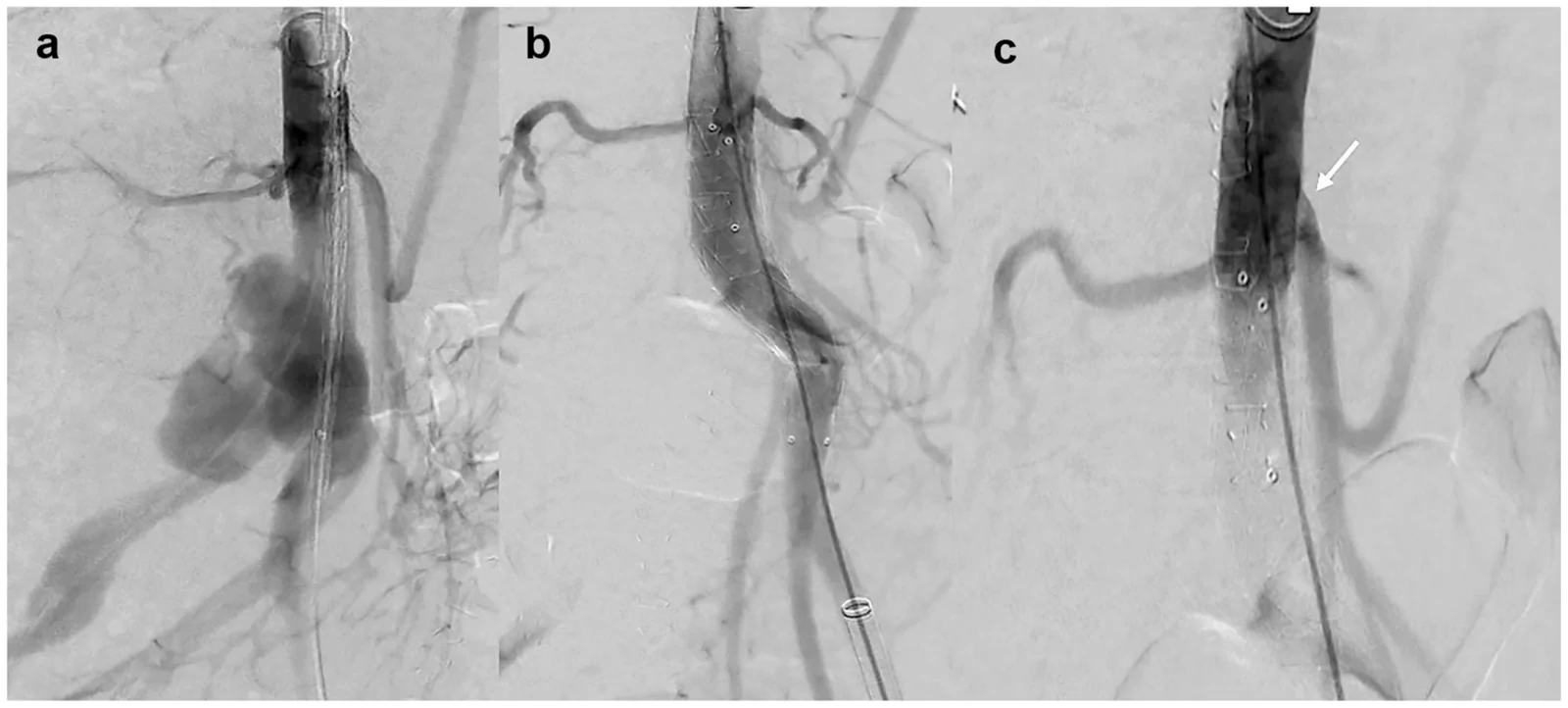
**a,** Preoperative aortogram demonstrating multiple pseudoaneurysms involving the abdominal aorta and bilateral iliac arteries. **b,** Deployment of an 16 × 13 × 93 mm Endurant II iliac limb endograft (Medtronic) extending from the infrarenal aorta, below the origin of the inferior mesenteric artery, to the left common iliac artery. **c,***White arrow* indicating a patent inferior mesenteric artery with the proximal end of the endograft positioned below its origin.

**Fig 5:**
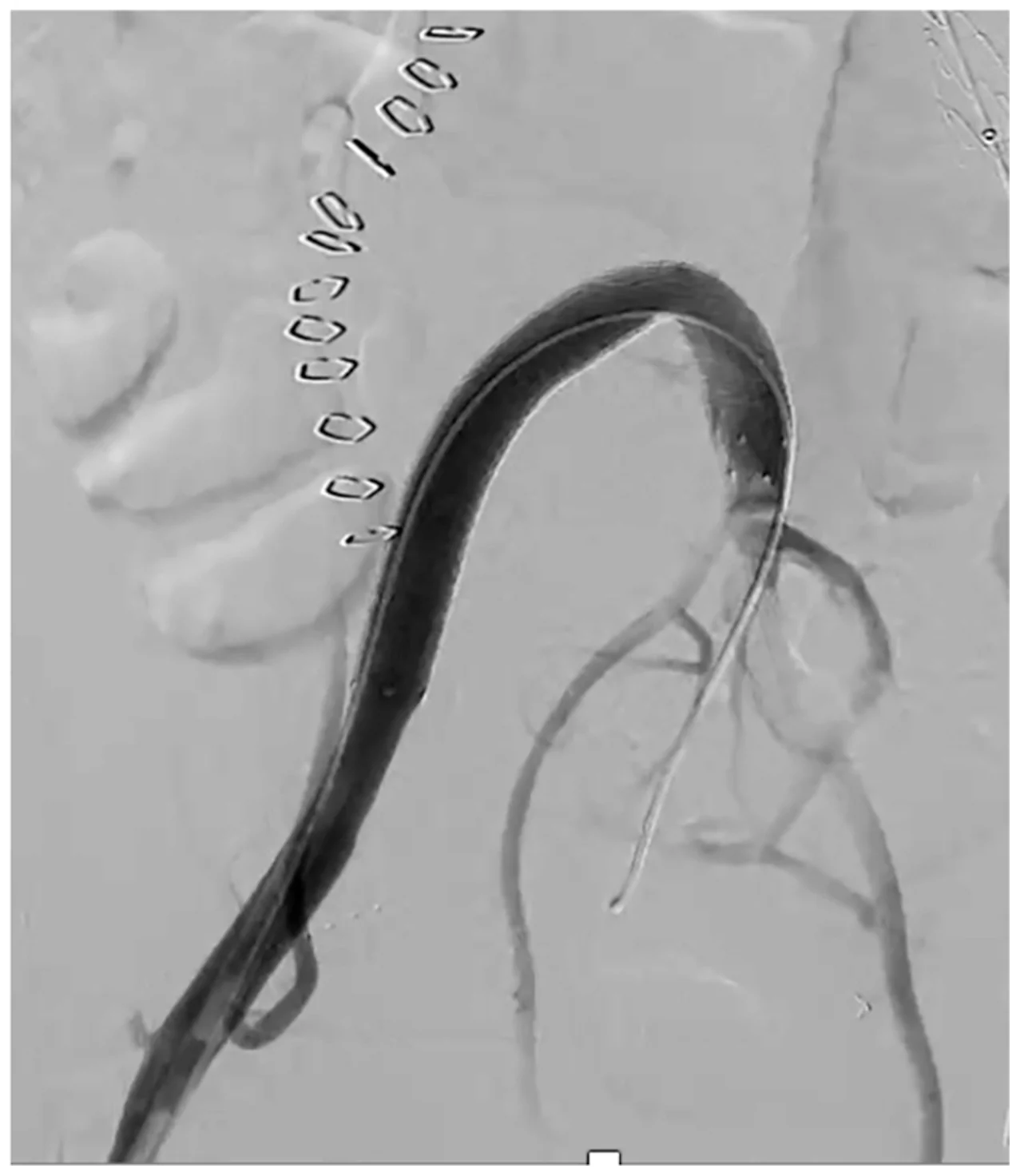
The banana technique using an 11 mm × 10 cm VIABAHN covered stent (W. L. Gore & Associates) to preserve perfusion to the right internal iliac artery while excluding the right common iliac artery pseudoaneurysm.

The patient was discharged on postoperative day 14 on aspirin 81 mg daily and ongoing neurosarcoidosis therapy including prednisone, infliximab, and methotrexate. At the 6-month follow-up, the patient had regained partial neuromuscular function and is able to ambulate with a walker while continuing rehabilitation. The patient remained on immunosuppressive therapy and under the care of the neurology and rheumatology teams. All vascular reconstructions and revascularization procedures remained patent without evidence of recurrent aneurysmal degeneration, endoleak, or any evidence of graft infection.

## Discussion

Sarcoidosis is a chronic inflammatory granulomatous disorder characterized histologically by noncaseating granulomas. Although pulmonary involvement predominates, extrapulmonary manifestations may affect virtually any organ system. Neurosarcoidosis is among the most severe extrapulmonary manifestations and may involve the meninges, spinal cord, cranial nerves, or peripheral nervous system.^2^^,^^4^ Vascular involvement in sarcoidosis is uncommon but increasingly recognized, particularly in the form of large-vessel vasculitis and aortitis. The pathophysiology of vascular sarcoidosis is thought to involve granulomatous infiltration of the arterial wall, leading to destruction of elastic fibers and medial degeneration.^3^ Progressive weakening of the vessel wall may subsequently result in aneurysm formation, pseudoaneurysm degeneration, stenosis, or occlusive disease. Both thoracic and abdominal aortic involvement have been described, although visceral artery aneurysms associated with sarcoidosis remain exceedingly rare.^5^, ^6^, ^7^

The present case is notable for several features suggestive of systemic vascular involvement, including the patient’s young age, multifocal aneurysmal disease affecting multiple vascular territories, concomitant neurosarcoidosis, and absence of a known hereditary connective tissue disorder. Differential diagnoses included vascular Ehlers-Danlos syndrome, Loeys-Dietz syndrome, segmental arterial mediolysis, and infective (mycotic) aneurysms. Infectious aneurysmal degeneration remained an important consideration given the patient’s recurrent fevers and immunosuppressive therapy. Although FDG-PET demonstrated no increased metabolic activity within the aneurysmal segments, a false-negative result could not be entirely excluded, particularly in the setting of immunosuppression. Consequently, graft selection was based on a comprehensive multidisciplinary assessment integrating the clinical presentation, serial microbiological investigations, imaging findings, and anatomical considerations, rather than relying solely on FDG-PET findings.

The morphology and distribution of the aneurysms also posed substantial technical challenges. The tandem SMA aneurysms extended distally beyond the proximal vessel, involving critical mesenteric branches without an adequate distal landing zone for covered stent exclusion. Consequently, isolated endovascular repair was not anatomically feasible. Hybrid visceral revascularization permitted preservation of distal mesenteric perfusion while allowing endovascular exclusion of the aneurysmal segments.

This case further highlights the complexity of managing vascular pathology in patients with systemic inflammatory disease and significant comorbid conditions. The patient’s concurrent renal cell carcinoma, immunosuppressive therapy, recurrent febrile episodes, neurologic disability, and social factors required careful coordination among multiple specialties. A staged operative strategy was therefore selected to minimize physiologic stress and reduce the risk of perioperative complications associated with combined extensive visceral and aortoiliac reconstruction.

Although vascular sarcoidosis remains rare, clinicians should consider inflammatory vasculitis in young patients presenting with multifocal aneurysmal disease involving atypical vascular territories. Advanced imaging modalities such as FDG-PET may assist in evaluating active vascular inflammation and excluding infectious etiologies. Given the limited literature regarding optimal management, treatment strategies must frequently be individualized according to anatomy, inflammatory activity, comorbidities, and patient condition.

In conclusion, this case describes a rare presentation of multifocal visceral and aortoiliac pseudoaneurysms in a young patient with neurosarcoidosis. The patient required complex staged hybrid vascular reconstruction because of extensive aneurysmal involvement and challenging mesenteric anatomy. Although vascular manifestations of sarcoidosis are uncommon, this case highlights the potential for systemic granulomatous disease to involve multiple arterial territories and emphasizes the importance of multidisciplinary management and individualized operative planning in these complex patients.

## Patient consent

Informed consent was obtained from the patient for publication of the case report and accompanying images.

## Funding

None.

## Disclosures

None.
